# Design and Thermal Comparison of Random Structures Realized by Indirect Additive Manufacturing

**DOI:** 10.3390/ma12142261

**Published:** 2019-07-13

**Authors:** Daniele Almonti, Nadia Ucciardello

**Affiliations:** Dipartimento di Ingegneria dell’Impresa “Mario Lucertini”, Università di Roma “Tor Vergata”, Via del Politecnico 1, 00133 Rome, Italy

**Keywords:** metal foam, additive manufacturing, open cell, thermal test, random structure

## Abstract

Additive manufacturing (AM) processes are used to fabricate three-dimensional complex geometries. There are several technologies that use laser or electron beam over metal powder beds. However, the direct AM processes have inconveniences such as specific set of materials, high thermal stress traced, high local energy absorbed, poor surface finish, anisotropic properties, high cost of material powder, and manufacturing with high-power beams. In this paper, an alternative process was developed. An indirect additive manufacturing (I-AM) combining a 3D print of castable resin and metal casting in order to obtain a cellular structure similar in shape to commercial metal foams but completely definable as design features was developed. Design of the cellular structure was made by the graphical algorithm editor Grasshopper^®^. Designed structures were realized by a lost-wax casting process and compared with commercial foam specimens by a system designed for this work. The designed metal foams showed a performance superior to that of commercial metal foam; in particular, the heat thermal coefficient of designed metal foams in the better case was 870 W/m^2^·K, almost doubled in comparison with the commercial foam tested in this work.

## 1. Introduction

Additive Manufacturing (AM) is able to fabricate parts characterized with complex geometry from a three-dimensional Computer-Aided Design (CAD) system. AM components were made from 3D printing CAD files by adding material in subsequent layers [1,2]. There are various AM processes, and the differences among them are the way layers are realized to create parts, the operating principle, and the materials employed. Reference standard for AM processes is ISO/ASTM 52900:2015 (ASTM F2792) Additive manufacturing—General principles—Terminology. Fused Deposition Modeling (FDM) is a Material Extrusion (ME) process of a thermoplastic polymer. It uses a movable head, which deposits material into a substrate. Due to temperature control of the process, the polymer solidifies after extrusion and welds to the previous layer. FDM process is characterized by its cheapness, nontoxicity, and the different variety of materials, colors, and types that can be used, such as acrylonitrile butadiene styrene (ABS), medical ABS, poly(lactic acid) (PLA), investment casting wax, and elastomers [3,4]. FDM is appreciated by hobbyists for the production of low-cost plastic parts [5]. The process is characterized by simplicity, the relatively cheap equipment, and the raw materials. However, accuracy and surface quality are relatively poor compared to those of other AM processes [6]. Stereolithography (SLA) is a vat photopolymerization process in which liquid photopolymer in a vat is selectively cured by light-activated polymerization. The ultraviolet (UV) laser solidifies a thin layer on the working area. An SLA machine is composed of a working platform, which is immersed into a liquid resin, and a laser source that realizes the localized polymerization. The AM process that allows the realization of metal parts is a very interesting technique for industrial and aerospace application. The most diffused processes for metals AM are divided into direct energy deposition process, in which focused thermal energy is used to fuse materials by melting while deposited, and powder bed fusion, in which thermal energy selectively fuses regions of the powder bed. These processes compared to AM with polymeric materials are more interesting for structural application but the expensive machines, the difficulty of setting parameters, and the low size of produced parts limit their industrial applications [7]. Thanks to the possibility of realizing complex geometry without design limits, AM technologies represent an interesting process to realize metal foams. Metal foams are receiving attention due to their combinations of various thermal, mechanical, and acoustic properties that provide potential opportunities for diverse multifunctional structural applications [8,9]. In particular, they have high specific strength [10,11,12,13,14], high specific strain [15,16,17], excellent impact absorption [18,19], acoustic insulation [20], and compact heat exchangers [21,22,23,24,25,26]. The main problems related to their production are the control of porosity distribution [27,28] and the assembly of foams with other components. The connection between metal foam and the support element is fundamental for structural and heat exchangers applications. However, welding and brazing are time-consuming and expensive; for these reasons their application into industrial manufacturing processes is hard [29,30]. The design of the cellular structure has been fundamental because the purpose of the design process is to define a morphology, completely definable and repeatable, of the structure of commercial metal foam that can be modified according to the specific application. Due to their random structure, the commercial foams are very interesting for application as a filter [31,32,33] or heat transfer [34,35]. The aim of this work is the design, realization, and thermal comparison of structures similar to commercial foams, but, due to AM technology, definable in the various geometrical characteristics as sections of ligaments and pore distribution. 

## 2. Materials and Methods 

**Figure 1:**
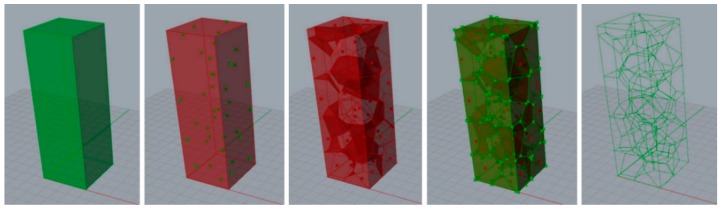
Procedure to obtain random structure from a space region by Voronoi tessellation.

The cellular structure was designed by Spatial Voronoi Tessellation by using Rhinoceros’ plug-in GrassHopper. A volume corresponding to specimen was populated with a random cloud of points corresponding to cell cores. The number of points in function of the number of PPI (pores per inch) that designed metal foam was determined. A 3D Voronoi tessellation is a division of a domain in 3D space, *D* ∈${\mathbb{R}}^{3}$, into a distribution of cells. Defined a number of points in *D*, $\left\{S_{i}\left(x_{i}\right)\right\}$ for i={1,…,N}, every point is associated a Voronoi cell, $C_{i}$, as follows:

(1) $$C_{i}=\left\{P\left(x\right)\in D|d\left(P,S_{i}\right)\leq d\left(P,S_{j}\right)\;\;\forall j\neq i\right\},$$

where *d*(⋆,⋆) is the Euclidean distance. The point positions are randomly chosen from a uniform distribution in the domain. The defined cell can be seen as the influence region of a point, which is the region of domain closer to the point. There are various proposed algorithms for realizing Voronoi tessellations. In this work, a cell-by-cell construction algorithm was deployed. A cell ($C_{i}$ of point $S_{i}$) is first set as the whole region. $C_{i}$ is then modified by an iterative process, where other points ($S_{j}$) are considered by increasing distance from $S_{i}$. At each iteration (point $S_{j}$), $C_{i}$ is reduced to the intersection of the previously computed cell and the half-space closer to $S_{i}$ than to $S_{j}$. This iterative process can be stopped when the distance between point $S_{i}$ and point $S_{j}$ becomes high enough for the half-space closer to $S_{i}$ than to $S_{j}$ to necessarily incorporate the whole cell. Equation (2) shows the isotropic criterion:

(2) $$\{\begin{array}{c} d\left(S_{j},S_{i}\right)>2d_{max}\\ d_{max}=max_{P\in C_{i}}d\left(P,S_{i}\right)=max_{P\in \left\{V_{i}\right\}}d{\left(P,S_{i}\right)}_{i} \end{array},$$

where {*V_i_*} is the collection of vertices of cell *C_i_*. The resulting set of cells fully defines the spatial tessellation. Voronoi cells are convex polyhedra intersecting along flat faces, straight edges, and vertices. Figure 1 shows the tessellation process that has been adopted to obtain foam structure. The region of space object of the tessellation has been defined; subsequently, this region has been populated with a random distribution of points representing the cell cores. These points were used to identify the Voronoi cells and then the lattice structure used to obtain the CAD model of the foams was identified. 

The pore densities commonly used in the industrial field are those of 5 and 10 PPI, so, in addition to these two densities, it has been decided to create an intermediate of 7 PPI. The specific number of pores was calculated in Table 1.

**Figure 2:**
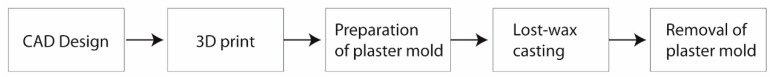
Scheme of process to realize metal foam designed in this paper.

The pore size distribution is considered a significant structural parameter despite the total porosity [36]. Voronoi tessellation allows individuating for each core of the random distribution, the regions of space by considering the points that are less distant from each core than the others. For each fixed core, the axial planes of the segments that join it to the nearby cores identify a convex cell. Once the reticular structure was made, a thickness of 0.4 mm was applied to obtain ligaments according to commercial foams’ geometric characteristics. In order to realize the printable model, the CAD file was cleaned from open surfaces and repeated entities. In the intersection between segments, connecting rays were added to obtain a foam structure similar to the commercial one. The foundry model was realized by a stereolithography 3D printer XFAB 2000 DWS. This 3D printer presents a cylindrical work area of 180 mm and it uses a laser source, Solid State BluEdge^®^ (Thiene, Italy) BE-1300X, that allows obtaining slice thickness in a range of 10–100 µm, and it can realize minimum feature size pair as 250 µm. The material used, FUSIA 444^®^, is a photosensitive material for DWS Stereolithography 3D printers, suitable for direct casting of detailed thin and solid models (Table 2). Thanks to the reticular structure of the foam, it was not necessary to add supports for the correct realization of the obtained components. The printed models were placed in a UV oven for 1 h in order to perfectly complete the polymerization. Figure 2 shows scheme of process to realize metal foam designed in this paper. 

**Figure 3:**
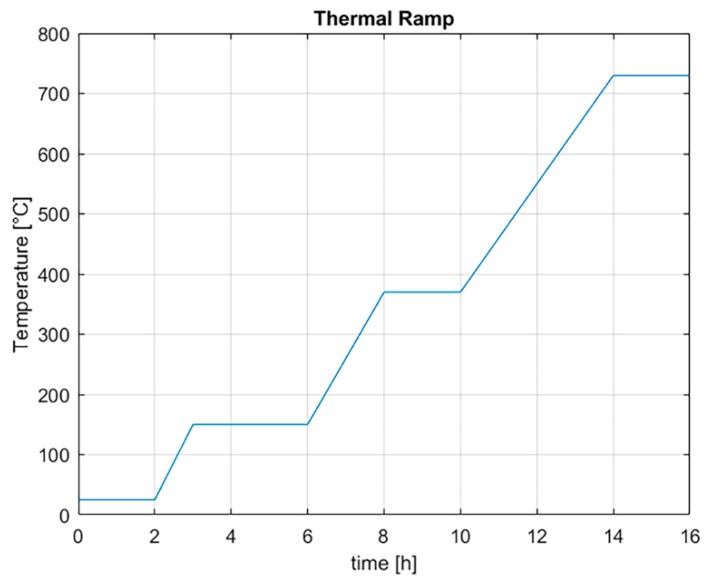
Thermal ramp to prepare the plaster for melting process.

The master model obtained was used in a process similar to an indirect rapid tooling that allows reducing lead times compared to conventional tooling manufacturing. By the previous process, the sacrificial pattern to use in the realization of mold plaster (Ultra-Vest^®^ MAXX, Ransom & Randolph, Maumee, OH, USA) was obtained. To produce the plaster suspension, water at 25 °C was used. First, the plaster was added to the water, left to soak for 1 min, and then mixed for 4 min at 316 rpm. The plaster suspension was poured into the foundry cylinder to form the plaster mold. The plaster preparation was done by using a vacuum machine (SL4^®^, Mario Di Mario, Milano, Italy) and a vibrating platform to avoid air inclusions that cause defects on the final shape. The plaster mold was dried at room temperature for 2 h, after the dewaxing process was done. The plaster mold was heated up to 150 °C in 1 h, maintained at this temperature for 3 h, then heated up to 370 °C in 2 h, maintained at this temperature for 2 h, then finally, heated up to 730 °C, and it remained at this temperature for 2 h. This thermal procedure was fundamental to eliminate the resin from the mold and reach the temperature of casting. The temperature ramp for carrying out the melting process is shown in Figure 3.

Meanwhile, the aluminum alloy EN43500 (AlSi10MnMg) was heated up to casting temperature at 730 °C and mixed by low-frequency pulses. To realize the casting process, a casting machine operating under vacuum was used that could reach the maximum temperature of the casting of 1450 °C with an accuracy of 4 °C due to multiple thermocouple checks. After the casting process, the cylinder was cooled for 6 h and then the plaster was removed. The metal foam samples obtained were removed from the sprues and the pouring channels and were brought to the dimensions of 20 × 20 × 40 mm^3^; the specimen with similar dimensions was obtained from commercial metal foam, Duocel^®^ by ERG Aerospace Corporation (Oakland, CA, USA). The commercial foam used in this work was fabricated by pre-forming the polymer foam. The commercial metal foams that were used in this work were each 10 PPI obtained with the same material of the designed samples. Porosity and transfer surface of the specimens were characterized. The porosity defined in the following equation was derived by the relative density ($\rho^{*}/\rho$)

(3) $$\varepsilon =1-\frac{\rho^{*}}{\rho },$$

where $\rho^{*}$ is the density derived from the proportion between the mass and the volume individuated in the CAD environment, and ρ is the density of the bulk material. Thanks to the CAD models, transfer surface areas were measured. Successively, thermo-fluid analysis was involved to determine convective Heat Transfer Coefficient (HTC*) and Pressure Drop (PD). 

To identify the thermal behavior and make a comparison between the commercial model and the designed foams, a specific instrumentation was realized. The instrumentation consisted principally of two stainless steel (AISI 316) conduits characterized by square internal cross-section of 20.5 × 20.5 mm^2^ and length pair of 1000 mm. In the middle of the conduits, an aluminum cylinder was installed with a 40 mm long square section cavity. This component had the function of sample holder. It was attached to the ducts by means of screws. It had also the purpose of dividing the pipes and positioning along a longitudinal plane the specimens for the characterization. Some foils of mica and Bakelite were inserted between the cylinder and the pipes with the aim to minimize the heat flow in the axial direction, favoring the radial one. The air was pushed by the compressed air system, which was able to ensure dry air at different speeds. The air speed was controlled by an anemometer (AM-4204, Lutron Electronic, Milano, Italy) that was located in the supply conduit. Four thermocouples (K-type) were installed along the two ducts, two at the start and at the end of the tubes and the other two before and after the sample holder component. The thermocouples were linked to a data recorder (TC-08, Pico Technology, St Neots, UK). Thanks to PicoLog^®^ 5.25.3 software, temperature profiles were displayed and recorded. The PDs were quantified with a differential pressure gauge (PCE-P01, PCE Italia, Capannori, Italy) through two apertures directly before and after the foam space holder. Preliminary measurements were made in the absence of the specimens in order to determine the contribution of the apparatus on the PDs. A heating band of 230 W was positioned around the sample holder. Temperature of the sample was monitored by a digital controller (R-38, Ascon Technology, Vigevano, Italy). Thanks to the K thermocouple welded in the middle of the specimen, precise temperature control of the foam was achieved by a digital multimeter (Fluke 289, FLUKE, Everett, WA, USA). This new apparatus, unlike other existing systems, determines the HTC* using the energy balance between the heat transferred to the fluid Equation (4), and compares it to the one described by Newton’s law of convection, Equation (5):

(4) $$\dot{Q}=m\cdot \Delta h,$$

(5) $$\dot{Q}=HTC^{*}\cdot A\cdot \Delta T_{ml},$$

It is significant to emphasize that the HTC* determination followed a lumped parameter approach, and consequently, the HTC* was mediated on the whole sample. Specimens were lapped to the size of 20.5 × 20.5 × 40 mm^3^ to ensure a good contact between foam and sample holder. According to an experimental plan, a set of three measurements for each type of sample was performed. This plan was developed varying the speed of dry air in five levels (5.0, 7.5, 10.0, 12.5, 15.0 m/s).

## 3. Results and Discussion

**Figure 4:**
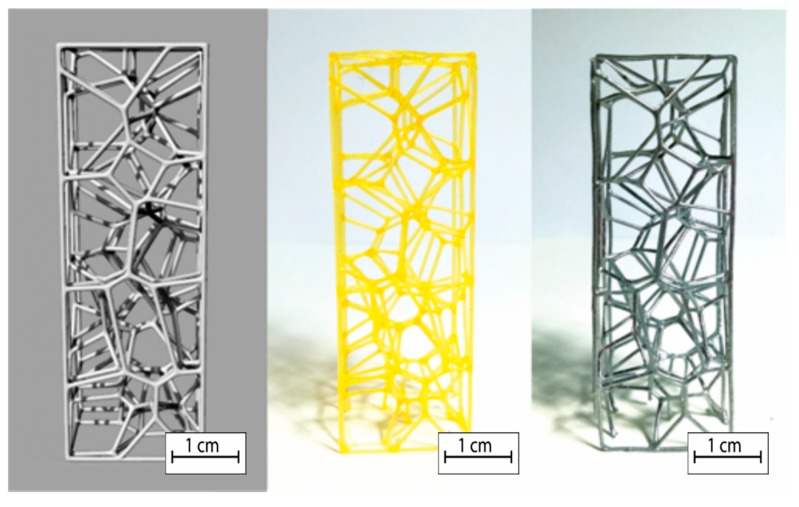
Comparison between CAD model, printed, and specimen obtained from foundry.

**Figure 5:**
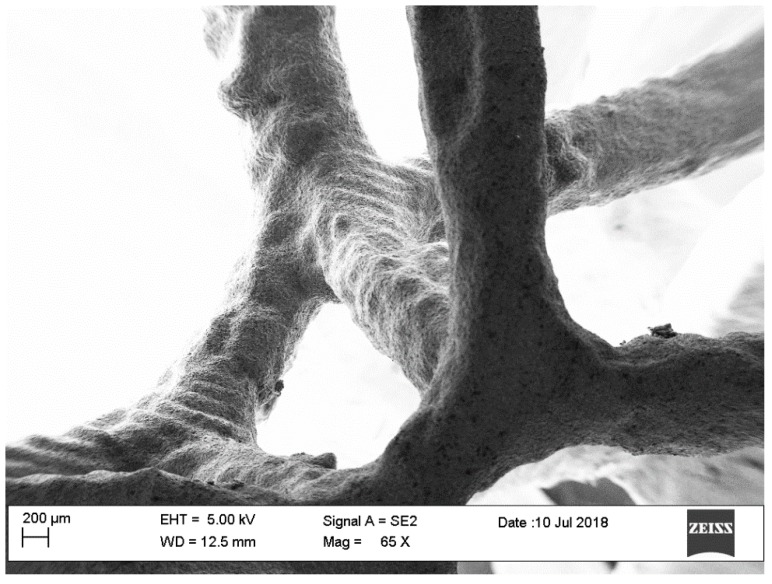
SEM image of 5 PPI designed metal foam.

Thanks to the elaborated algorithm, it was possible to realize a reticular structure completely designed with a random order of cell distribution very similar to the natural structure. Due to its particular shape, the structure is self-supporting, so during the additive process, it was not necessary to use supporting elements. The reticular structure obtained in this work shows exactly the number of cells required to be classified with the appropriate number of PPIs. The comparison between the CAD models and final specimens shows that geometric parameters are perfectly matching. In particular, the matching between the number of pores was made by macroscopic observations (Figure 4), while the check on section ligaments was made by SEM analysis that showed how the casting process generated the layer profile typical of the printing process (Figure 5). The SEM analysis showed the dimensional difference in the order of 0.4–0.6% in accordance with linear shrinkage due to solidification of the casting. In the melting process, as the structure thickened and therefore the number of PPIs increased, the fusion cavity was filled without damaging the gypsum shape (as expected from the thermal cycle employed, which allowed the material to not be viscous, and cooling that prevented the total filling of the form). The removal of the plaster from the structures obtained was carried out without difficulty.

Figure 4 shows how the structure obtained from the casting process is exactly the same as that obtained in the software environment. This highlights the complete control on parameters such as the number of cells and ligament dimensions. 

The casting process allowed obtaining a structure that replicates the printed model; in fact, Figure 5 shows the silhouette of the printing layer on the casted specimen. 

**Figure 6:**
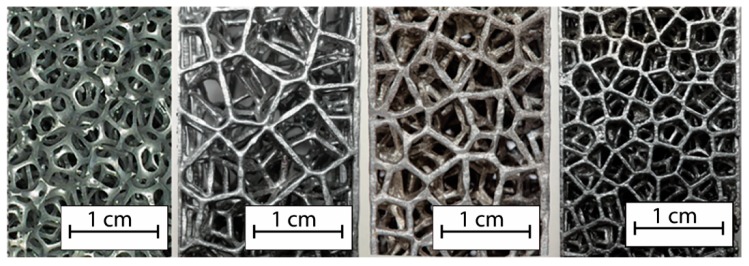
Metal foam objects of this work, from left to right, Duocel^®^, 5 PPI designed, 7 PPI designed, and 10 PPI designed.

Due to an accurate design operation of the random structure similar to the structure of commercial foams, samples having PPI number equal to the theoretical ones were obtained. The classification in PPI of the commercial models is a statistical classification as observed in the two commercial foams, the objects of the comparison; the structures are very different but both are marketed as 10 PPI foams. This depends on the process that is used to obtain the foams, since this is a partial control of the structure and the overall quality is obtained only thanks to a statistical approach [37]. Figure 6 shows the metal foam objects of this work. From left to right, the Duocel^®^ commercial foams and the following designed structures can be seen.

**Figure 7:**
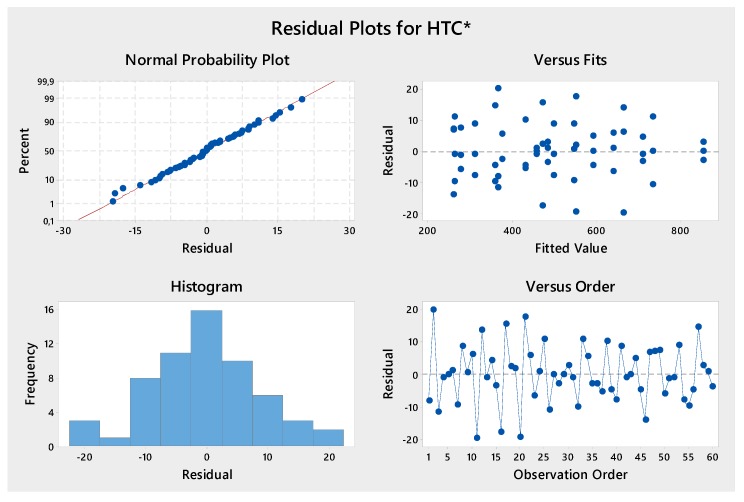
Residual plots for HTC*.

**Figure 8:**
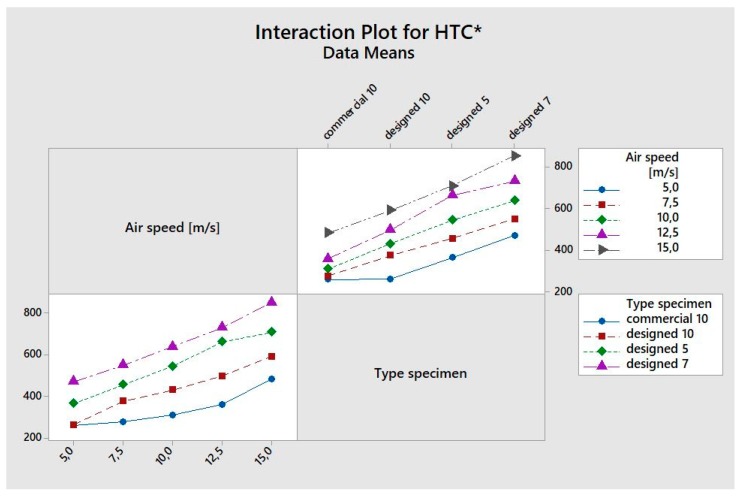
Interaction plot for HTC*.

Analysis of Variance (ANOVA) was performed to evaluate the significant iteration between the air speed value and typology of the specimen. The analysis was performed by using the software Minitab and considering the factors in Table 3. A general factorial regression of HTC* and pressure drop versus air speed and typology of specimen have been done. The results of ANOVA are shown in the following Figures and Table 4 in terms of *p*-value, F-value, and R-squared coefficients. By analyzing the p-value and assuming a confidence level of 95%, the two parameters and their iteration are significant (*p*-value < 0.05). The analysis of F-value suggests that the factor typology of specimen was the most significant. In Figure 7, the residual plots are reported; they do not reveal any model inadequacy or unusual problem. By analyzing the interaction plot in Figure 8, it shows how the two factors examined are independent of each other.

**Figure 9:**
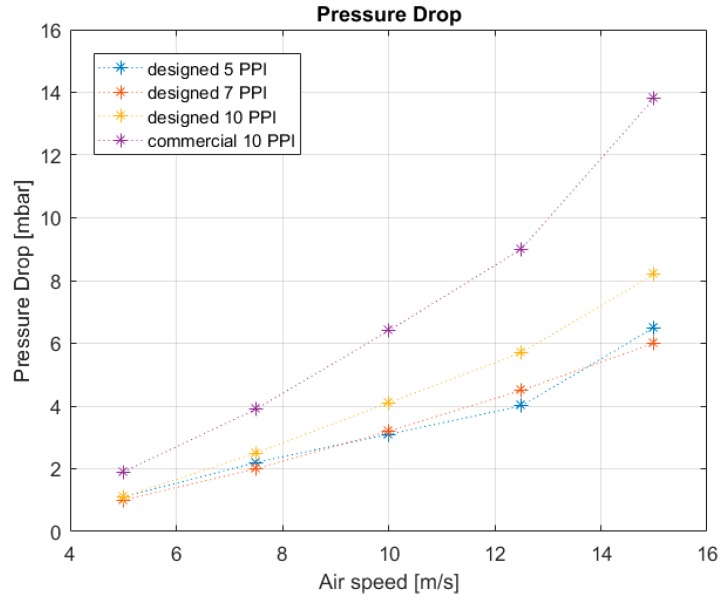
Pressure drop versus air speed of specific typology and comparison between the different models.

Figure 9 shows the trend of PD according to air speed for three foam pore sizes, 5, 7, and 10 PPI. As can be seen from Figure 9, the magnitude of PDs intensifies with air speed. A relevant growth was observed with a 10 PPI commercial foam. The designed foam trends are in accordance to the one obtained; in particular, the 5 and 7 PPI foams have similar behaviors. This peculiar trend can be attributed to the morphology of the samples, in particular, size and pore distribution. Specimens composed of 10 PPI foams did not trigger significant turbulence phenomena in the studied air flow. The morphology of commercial foam limits the passage of air and therefore favors convective heat exchange with respect to the designed models. The 7 PPI commercial foam results were characterized by higher turbulence status. In order to verify the repeatability of the measurements, each type of test was repeated three times. The results obtained from these first campaign test runs showed that the test facility can be considered reliable and the data repeatable. The achieved results are in accordance with literature [38]. Subsequently, for this previous campaign, using the same apparatus test, specimens of metal foams were thermally characterized. 

**Figure 10:**
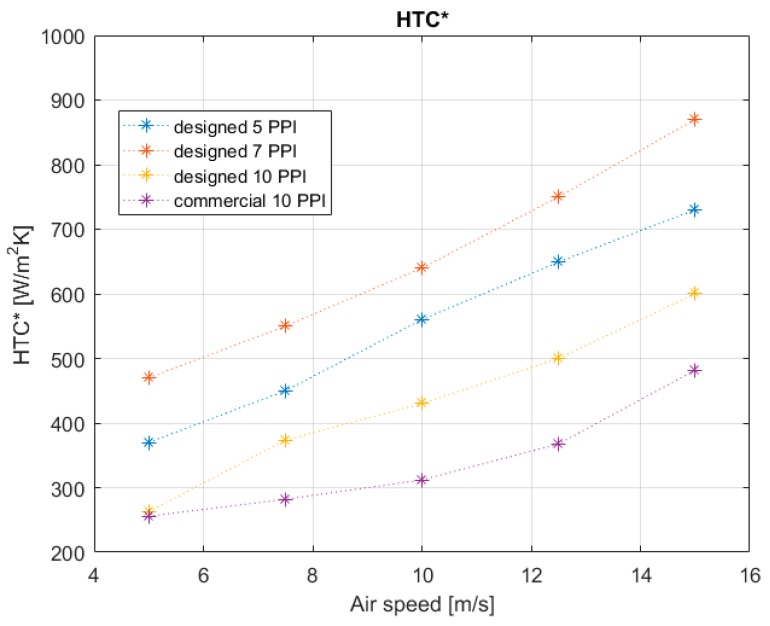
HTC* versus air speed of specific typology and comparison between the different models.

**Figure 11:**
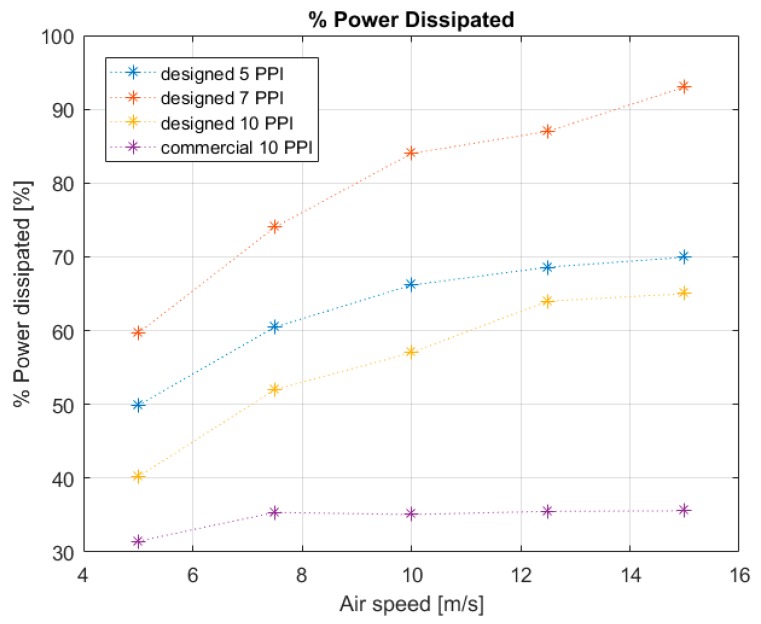
Percentage of power dissipated versus air speed of specific typology and comparison between the different models.

Figure 10 shows the heat transfer results. The global heat transfer coefficients for the four models are plotted against the air speed as a function of the imposed air mass flow rate. Experimental tests allowed studying the influence of geometric characteristics of the foam during the development of the thermal phenomena. The global HTC* of the different aluminum foams shows the same evolvement for the various imposed air speeds. The value of HTC* obtained for the commercial foam with an air speed pair to 5 m/s is in accordance with literature [21]. The results demonstrate that the HTC* rises with increasing air flow rate and it varies in a range between 256 and 870 W/m^2^·K depending on the PPI value. The higher value of HTC* obtained in this work was achieved by the 7 PPI foams (HTC* = 870 W/m^2^·K). Instead, commercial foam is characterized by lower thermal efficiency. Figure 10 shows that the 10 PPI aluminum foams present a global HTC* lower than 7 PPI aluminum foams and 5 PPI foams. 7 PPI and 5 PPI foams, despite having less exchange surface than 10 PPI foams, develop the best turbulence condition, promoting more efficient thermal exchange phenomena. The 10 PPI metal foams have the highest heat transfer surface. In fact, the transfer area increases as the number of PPI increases. Figure 11 shows the percentage of power dissipated thanks to the air flow though the foam structure. The 7 PPI designed structure is the best solution to dissipate the power generated from the thermal band; in fact, this configuration dissipated the maximum percentage with respect to other specimens of this work (93%). There is evidently a trend in the other specimens: the 5 PPI metal foams dissipated power in function of their surface area transfer, while the 10 PPI metal foams (both commercial and designed) showed an elevated pressure drop that did not allow fluid passage and, consequently, thermal dissipation.

In this study, only one ligament section size was considered (in accordance with the commercial foam compared). Possible future studies can be:

- Observing the feasibility of the geometric characteristics regarding both the printing process and the subsequent casting process.
- Study of thermal and mechanical characterization varying the section of ligaments in correlation with PPI value.
- Study of conduction phenomenon and convective phenomenon inside these structures separately.
- Realization of heat management system with integrated metal foams directly from the manufacturing process without welding and brazing that represents critical technologies for this class of materials.

## 4. Conclusions

In this work, metal foams with completely defined structures were designed and realized. An algorithm that allows realization of a Voronoi tessellation in a specific region was implemented. The CAD models were designed with defined number of pores and dimension of ligaments. The CAD models were used to realize the foundry model by using 3D printing. The comparison between the CAD model, the printed component, and the final specimen was done by demonstrating that it is possible to realize completely definable metal foam with this hybrid additive manufacturing process. The samples obtained were compared with commercial metal foam by an apparatus designed ad hoc. The influence of operative parameters, air speed, and pore size on the HTC* and on the Pressure Drop upon thermal behavior of metal foam was investigated. Thermal properties of the foams were individuated thanks to a full experimental campaign. The results demonstrate that the HTC* increases with increasing air flow rate and it varies between 256 and 870 W/m^2^·K depending on the PPI value. The 7 PPI foam shows the best thermal performance. Despite having less exchange surface than 10 PPI foam, it triggers significant turbulence condition, facilitating a more efficient thermal exchange phenomenon.

## Figures and Tables

**Table 1:** Number of pores of foams designed in this work.

PPI	Pores Per Centimeters	Specific Pores (Pores/cm^3^)	Numbers of Pores
5	1.968	7.628	183
7	2.756	20.931	502
10	3.937	61.024	1465

**Table 2:** Characteristics of photopolymer FUSIA 444^®^.

Technical Characteristics of the Liquid Material	
**Environmental Values for Use**	22–27 °C—max RH 40–60%
Appearance/Color	Liquid/Yellow
Viscosity	300–600 MPa·s at 25 °C
Density	1.08 g/cm^3^
**Technical Characteristics of the Resin after UV Curing**	
Surface Hardness	56–63 Shore D
Flexural Strength	10–16 MPa
Flexural Modulus	240–270 MPa
Elongation at Break	6–11%
Tensile Strength	6–13 MPa

**Table 3:** ANOVA: factors and levels.

Factor	Levels	Values	Unit
Air speed	5	5.0–7.5–10.0–12.5–15.0	m/s
Typology of specimen	4	commercial 10—designed 5—designed 7—designed 10	-

**Table 4:** Results of ANOVA: p-value, F-value, and R-squared coefficients.

Source	DF	Adj SS	Adj MS	F-Value	*p*-Value
Air speed (m/s)	4	751,416	187,854	1681.62	0.000
Typology of specimen	3	830,803	276,934	2479.05	0.000
Air speed (m/s) × Typology of specimen	12	47,774	3981	35.64	0.000
Error	40	4468	112		
Total	59	1,634,461			
